# A complete MAP kinase cascade controls hyphopodium formation and virulence of *Verticillium dahliae*

**DOI:** 10.1007/s42994-023-00102-y

**Published:** 2023-05-02

**Authors:** Ziqin Ye, Jun Qin, Yu Wang, Jinghan Zhang, Xiaoyun Wu, Xiangguo Li, Lifan Sun, Jie Zhang

**Affiliations:** 1 State Key Laboratory of Plant Genomics, Institute of Microbiology, Chinese Academy of Sciences, Beijing, 100101 China; 2 CAS Center for Excellence in Biotic Interactions, University of Chinese Academy of Sciences, Beijing, 100049 China; 3 State Key Laboratory of Crop Stress Biology for Arid Areas, College of Plant Protection, Northwest A&F University, Yangling, 712100 China; 4 School of Life Sciences, Hebei University, Baoding, 710023 China; 5 College of Agronomy, Shanxi Agricultural University, Taigu, 030801 China

**Keywords:** *Verticillium*, MAPK, Hyphopodium, Pathogenicity

## Abstract

Phytopathogens develop specialized infection-related structures to penetrate plant cells during infection. Different from phytopathogens that form appressoria or haustoria, the soil-borne root-infecting fungal pathogen *Verticillium dahliae* forms hyphopodia during infection, which further differentiate into penetration pegs to promote infection. The molecular mechanisms underlying the regulation of hyphopodium formation in *V. dahliae* remain poorly characterized. Mitogen-activated protein kinases (MAPKs) are highly conserved cytoplasmic kinases that regulate diverse biological processes in eukaryotes. Here we found that deletion of *VdKss1*, out of the five MAPKs encoded by *V. dahliae*, significantly impaired *V. dahliae* hyphopodium formation, in vitro penetration, and pathogenicity in cotton plants. Constitutive activation of MAPK kinase (MAPKK) VdSte7 and MAPK kinase kinase (MAPKKK) VdSte11 specifically activate VdKss1. Deletion of *VdSte7* or *VdSte11* resulted in a phenotype similar to that of the mutant with *VdKss1* deletion. Thus, this study demonstrates that VdSte11-VdSte7-VdKss1 is a core MAPK cascade that regulates hyphopodium formation and pathogenicity in *V. dahliae*.

**Supplementary Information:**

The online version contains supplementary material available at 10.1007/s42994-023-00102-y.

## Introduction

In eukaryotes, mitogen-activated protein kinase (MAPK) cascades are highly conserved cytoplasmic kinases that integrate extracellular signaling and transduce to downstream substrates to regulate a wide range of biological processes (Bigeard and Hirt 2018; Dixon et al. 1999; Gustin et al. 1998). The MAPK cascades consist of MAPK kinase kinases (MAPKKKs, MAPK/extracellular signal-regulated kinase (ERK)-kinase kinases/MEKKs), MAPK kinases (MAPKKs, MAPK/ERK- kinases/MEKs), and MAPKs that are sequentially phosphorylated (Widmann et al. 1999). In general, MAPKKKs phosphorylate Ser and/or Thr residues located within the activation loop of MAPKKs, which in turn trigger dual phosphorylation of a highly conserved TXY motif located within the activation loop of MAPKs to activate MAPKs (Bigeard and Hirt 2018; Dixon et al. 1999; Gustin et al. 1998; Segmuller et al. 2007).

In fungi, MAPKs regulate growth and development, maintenance of cellular integrity, and responses to stress (Gustin et al. 1998; Jiang et al. 2018; Wang and Dohlman 2002; Zhao et al. 2005). In pathogenic fungi that infect plants, MAPK cascades regulate the pathogenesis and development of infection-related structures (Jiang et al. 2018; Li et al. 2019; Zhao et al. 2005). For instance, in the ascomycete *Magnaporthe oryzae*, which infects rice, barley, and other crops causing disease, pathogenicity MAP kinase 1 (Pmk1) is crucial for appressorium formation. MEKK MST11-MEK MST7-MAPK PMK1 constitutes a MAPK cascade regulating infection-related morphogenesis (Jiang et al. 2018; Zhao et al. 2005). In *Colletotrichum gloeosporioides*, Cgl-Slt2 MAPK plays an important role in the early stage of appressorium formation (Yong et al. 2013).

*Verticillium dahliae* infects a broad range of plants and causes severe wilt disease and agricultural losses. The development of infection-related structure is crucial for successful infection of host plants. Unlike *M. oryzae* and *C. gloeosporioides*, which form appressoria during infection, *V. dahliae* adheres tightly around the plant root surface and forms hyphopodia, which further develop into penetration pegs during infection (Zhao et al. 2014; Zhou et al. 2017). This process involves calcium accumulation, NADPH oxidase-dependent reactive oxygen species burst, and cAMP-mediated signaling (Sun et al. 2019; Zhao et al. 2014; Zhou et al. 2017); however, additional signaling components as well as the molecular mechanisms regulating these events remain elusive. The MAPKKK VdSte11, but not VdSsk2, plays a major role in regulating *V. dahliae* penetration (Yu et al. 2019). However, the core MAPK and complete MAPK cascade regulating hyphopodium formation in *V. dahliae* remain uncharacterized.

In this study, we investigate the functions of MAPKs, MAPKKs, and MAPKKKs in hyphopodium development in *V. dahliae*. Mutations in *VdKss1*, *VdSte7*, or *VdSte11*, respectively, significantly disrupted hyphopodium formation, in vitro penetration, and pathogenicity in cotton plants. The constitutive active forms of VdSte11 and VdSte7 specifically induced the phosphorylation of VdKss1. Thus, VdSte11-VdSte7-VdKss1 constitutes a complete MAPK cascade that regulates hyphopodium formation and pathogenicity of *V. dahliae* in host plants.

## Materials and methods

### Fungal strains, plant materials and culture conditions

Colonies of *V. dahliae* V592 (Gao et al. 2010) strain, *HopAI1*-expressing strain and mutation strains of target genes generated in this study were routinely cultured on potato dextrose agar (PDA) medium at 25 °C for 7 days in the dark. To harvest spores, hyphae block was cultured in potato dextrose broth (PDB) liquid medium with shaking at 150 rpm at 25 °C for 4 days. Cotton plants (‘Xinluzao No. 16’) were used in this study for virulence assessment (Zhou et al. 2017). All primers used in this study are listed in Table. S1 (see in Supplementary Information).

### Generation of the *HopAI1* expression construct and transformant

The *HopAI1* gene was amplified from *Pseudomonas syringae* DC3000 strain and cloned into the pSULPH-Tef-GFP vector to generate the pSULPH-Tef-*HopAI1*-GFP plasmid. The *HopAI1* expression construct then was transformed into *Agrobacterium tumefaciens* EHA105 strain, used for *Agrobacterium tumefaciens* mediated transformation (ATMT), as described previously, to generate the *HopAI1-*expressing transformant (Wang et al. 2016a).

### Generation of gene deletion mutants and complementary strains

To generate the target gene deletion plasmids, upstream and downstream genomic sequences of corresponding genes were amplified from *V. dahliae* V592 and cloned into the pGKO-HPT vector respectively. The resulting plasmids were used for ATMT to generate the deletion mutants (Wang et al. 2016a). To obtain the complementary strains of target genes, the genomic coding regions of target genes were PCR amplified and cloned into the pSULPH-Tef-GFP vector, respectively. The resulting constructs were transformed into *Agrobacterium tumefaciens* EHA105 strain respectively, and used for ATMT.

### Penetration assay and hyphopodium visualization

Sterilized cellophane membrane (DINGGUO, Beijing, China), used to simulate plant root surface to induce formation of hyphopodium, was covered on MM medium (Zhao et al. 2016). Equal amounts of conidia collected from *V. dahliae* strains as indicated were incubated on the cellophane membrane and grown at 25 °C for 5 days. The cellophane membrane was then removed and observed under the Leica SP8 confocal laser scanning microscope system for determining hyphopodium formation. Further culture for an additional 3 days to observe whether there were hyphae grew on the underlying medium.

### Pathogenicity assay

The conidia of *V. dahliae* strains as indicated were collected and resuspended in sterilized water at the concentration of 10^7^/mL. Cotton plants were infected by the root-dipping inoculation method (Gao et al. 2010). Cotton plants then were photographed and subjected to disease index analyses 3–4 weeks post inoculation. The disease index was classified as follows: 0 (no symptoms), 1 (0% – 25% wilted leaves), 2 (25% – 50% wilted leaves), 3 (50% – 75% wilted leaves) and 4 (75% – 100% wilted leaves). The disease index was calculated as 100 × [sum (number of plants × disease grade)]/[(total number of plants) × (maximal disease grade)] (Xu et al. 2014).

### Detection of MAPK phosphorylation

*V. dahliae* protoplasts were isolated from 36 h vegetative hyphae grown in CM medium and transfected with plasmid as described (Rehman et al. 2016). Fourteen hours after transfection, proteins of transfected protoplasts were extracted with extraction buffer containing 50 mM HEPES (4-(2-Hydroxyethyl)-1-piperazineethanesulfonic acid sodium salt) (pH 7.5), 150 mM KCl, 1 mM EDTA (ethylenediaminetetraacetic acid), 1 mM DTT (dithiothreitol), 0.2% Triton X-100, and 1 × proteinase inhibitor cocktail and subjected to IP and immunoblot assay with anti-pERK, anti-HA or anti-FLAG.

The presence of VdKss1-HA, VdMAPKKs-FLAG and VdMAPKKKs-FLAG was detected by anti-HA or anti-FLAG immunoblot. For anti-HA IP, total proteins were incubated with 2 µg of anti-HA antibody together with protein A agarose at 4 °C for 4 h. The agarose beads were collected and boiled for 5 min with 1 × protein loading buffer. Total proteins were separated in a 10% SDS-PAGE gel and transferred to the PVDF membrane. The phosphorylation of VdKss1 TEY motif was detected by anti-pERK immunoblot.

## Results

### Expression of a MAPK inhibitor compromises hyphopodium formation in *V. dahliae*

**Fig. 1:**
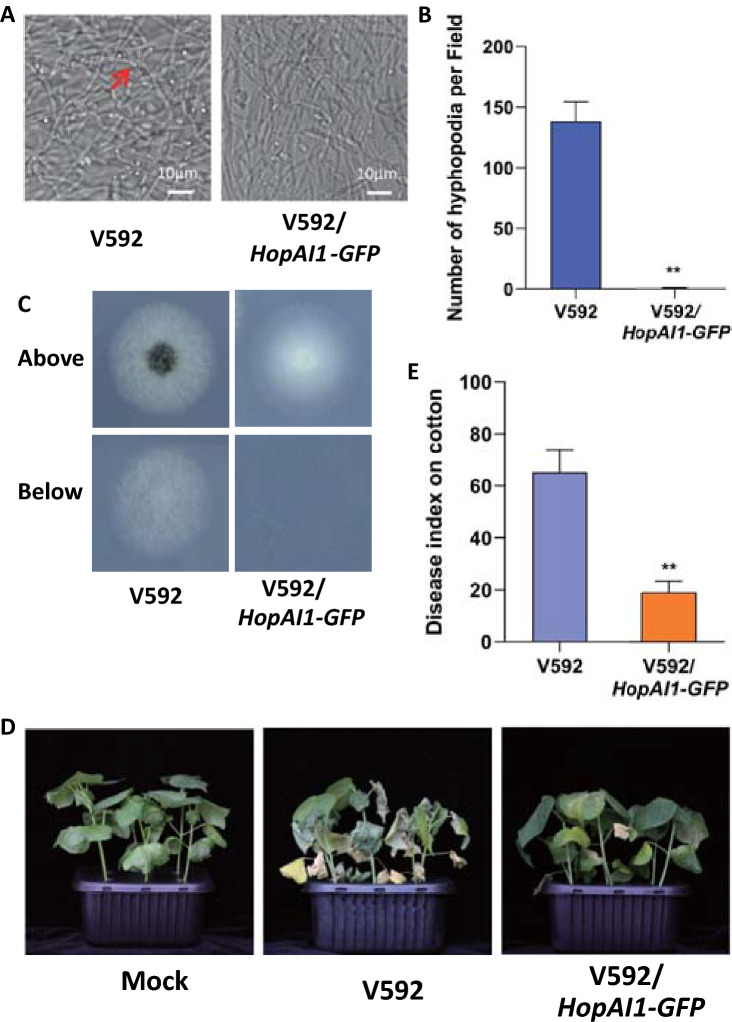
Expression of a MAPK inhibitor in *Verticillium dahliae* compromises hyphopodium formation. **A** Hyphopodium formation is suppressed by HopAI1. V592 and *HopAI1*-expressing strains were grown on MM covered with a cellophane for 5 days. Hyphopodium formation (red arrow) on the cellophane was observed by confocal laser scanning microscopy (CLSM). **B** Hyphopodium quantification of V592 and *HopAI1*-expressing strains. The number of hyphopodium in three fields was counted for each strain. Error bars indicate the standard deviation of three fields. Student’s *t*-test was carried out to determine the significance of difference. **Indicates significant difference at *P*-value of < 0.01. **C** The *HopAI1*-expressing strain displays a defect in penetration. The spores of V592 and *HopAI1*-expressing strains were grown on minimal medium (MM) covered with a cellophane for 5 days and photographed (above). The cellophane was removed and the culture was continued for 3 days and photographed (below). **D** Suppression of MAPK compromises *V. dahliae* virulence. Disease symptoms of upland cotton plants infected with the V592 and *HopAI1*-expressing strains were photographed and subjected to disease index analyses 3 weeks post inoculation. **E** Disease index analyses of upland cotton infected with the indicated strains. The disease indexes were evaluated with three replicates generated from 18 plants for each inoculum. Error bars indicate the standard deviation of three biological replicates. Student’s *t*-test was carried out to determine the significance of difference. **Indicates significant difference at *P*-value of < 0.01

MAPK cascades are conserved signaling kinases that regulate diverse biological processes. HopAI1, a *Pseudomonas syringae* effector protein, has a unique phosphothreonine lyase activity towards plant, animal and fungal MAPKs (Li et al. 2007a; Zhang et al. 2007, 2017). Expression of *HopAI1* in *M. oryzae* inhibits MAPK activation and appressorium formation (Zhang et al. 2017). To examine whether MAPK activation contributes to hyphopodium formation in *V. dahliae*, *HopAI1* was expressed in *V. dahliae*. The expression of *HopAI1* in the *HopAI1*-expressing strain was confirmed by anti-GFP immunoblot (Fig. S1). Similar to that in *M. oryzae*, expression of HopAI1 in *V. dahliae* can inhibit MAPK activity (Fig. S2). Compared with the wild-type (WT) *V. dahliae* strain, hyphopodium formation was significantly reduced in the *HopAI1*-expressing *V. dahliae* strain (Fig. 1A, B), suggesting that MAPK activation is crucial for *V. dahliae* hyphopodium development. WT and *HopAI1*-expressing *V. dahliae* strains were cultured on minimal medium (MM) covered with cellophane and examined for in vitro penetration (Zhao et al. 2016). Consistent with the deficiency of hyphopodium formation, the *HopAI1*-expressing strain displayed defects in in vitro penetration compared to the WT strain (Fig. 1C). WT and *HopAI1*-expressing *V. dahliae* strains were inoculated into cotton plants. Four weeks post inoculation, cotton plants infected with *HopAI1*-expressing strain exhibited much weaker disease symptoms than those infected with WT V592 (Fig. 1D). Disease index analyses indicated reduced pathogenicity of the *HopAI1*-expressing strain relative to the WT strain (Fig. 1E). These results strongly suggested that suppression of MAPK compromises hyphopodium formation and penetration, indicating an important role of MAPK activation in regulating hyphopodium formation of *V. dahliae*.

### Deletion of a specific MAPK compromises *V. dahliae* hyphopodium formation and penetration

Based on its homology with yeast homologs, *V. dahliae* encodes the following five putative MAPKs (Hamel et al. 2012): the Kss1 type VdKss1 (VDAG_09461), Hog1 types VdHog1-1 (VDAG_08982) and VdHog1-2 (VDAG_02354), Ime type VdIme2 (VDAG_06935), and Slt2 type VdSlt2 (VDAG_02584). To further confirm the requirement of MAPK activation for hyphopodium formation, *V. dahliae* mutants with gene deletion of individual *MAPKs* in the V592 WT strain were generated by homologous recombination (Fig. S3) using the ATMT-DS-vector (Wang et al. 2016a).

**Fig. 2:**
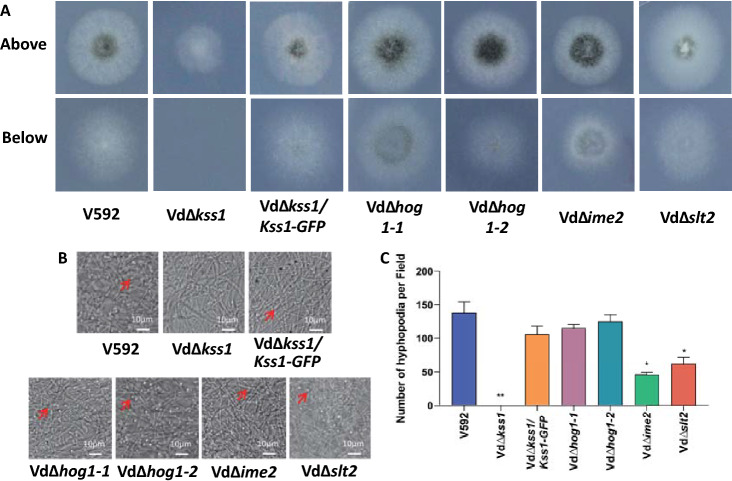
Deletion of a specific MAPK significantly compromises *Verticillium dahliae* hyphopodium formation and in vitro penetration. **A** Penetration phenotypes of *VdMAPKs* deletion mutants and Vd∆*kss1*/*VdKss1-GFP* complementary strain. The spores of indicated strains were grown on MM covered with a cellophane for 5 days and photographed (above). The cellophane was removed and the culture was continued for 3 days and photographed (below). **B** Hyphopodium formation of *VdMAPKs* deletion mutants and Vd∆*kss1*/*VdKss1-GFP* complementary strain. Indicated strains were grown on MM covered with a cellophane for 5 days. Hyphopodium formation (red arrow) on the cellophane was observed by confocal laser scanning microscopy (CLSM). **C** Hyphopodium quantification of *VdMAPKs* deletion mutants and Vd∆*kss1*/*VdKss1-GFP* complementary strain. The number of hyphopodium in three fields was counted for each strain. Error bars indicate the standard deviation of three fields. Student’s *t*-test was carried out to determine the significance of difference. *Indicates significant difference at *P-*value of < 0.05. **Indicates significant difference at *P-*value of < 0.01

The Vd*∆kss1*, Vd*∆hog1-1*, Vd*∆hog1-2*, Vd*∆ime2*, Vd*∆slt2*, and WT *V. dahliae* strains were grown on PDA medium, transferred to MM covered with cellophane, and tested for their ability to penetrate artificial membrane. At 4 days after growth, hyphal growth of all mutants and the WT strain, except for the Vd*∆kss1* mutant, was observed on the medium when the cellophane membrane was removed (Fig. 2A), suggesting that mutation of *VdKss1* compromised the capacity for membrane penetration. Next, we examined hyphopodium formation in these mutants. Mutation of *VdKss1* impaired hyphopodium formation (Fig. 2B, C). The complementation of* VdKss1* in the Vd*∆kss1* mutant restored membrane-penetration (Fig. 2A) and hyphopodium formation (Fig. 2B, C). Expression of VdKss1-GFP in the complementary strain was confirmed by anti-GFP immunoblot (Fig. S4A). Mutation of *VdIme2* and *VdSlt2* exhibited reduced hyphopodium formation (Fig. 2B, C), suggesting a contribution of *VdIme2* and *VdSlt2* to that process. These results indicated that *VdKss1* plays a major role, whereas *VdIme2* and *VdSlt2* play minor roles, in regulating hyphopodium formation.

**Fig. 3:**
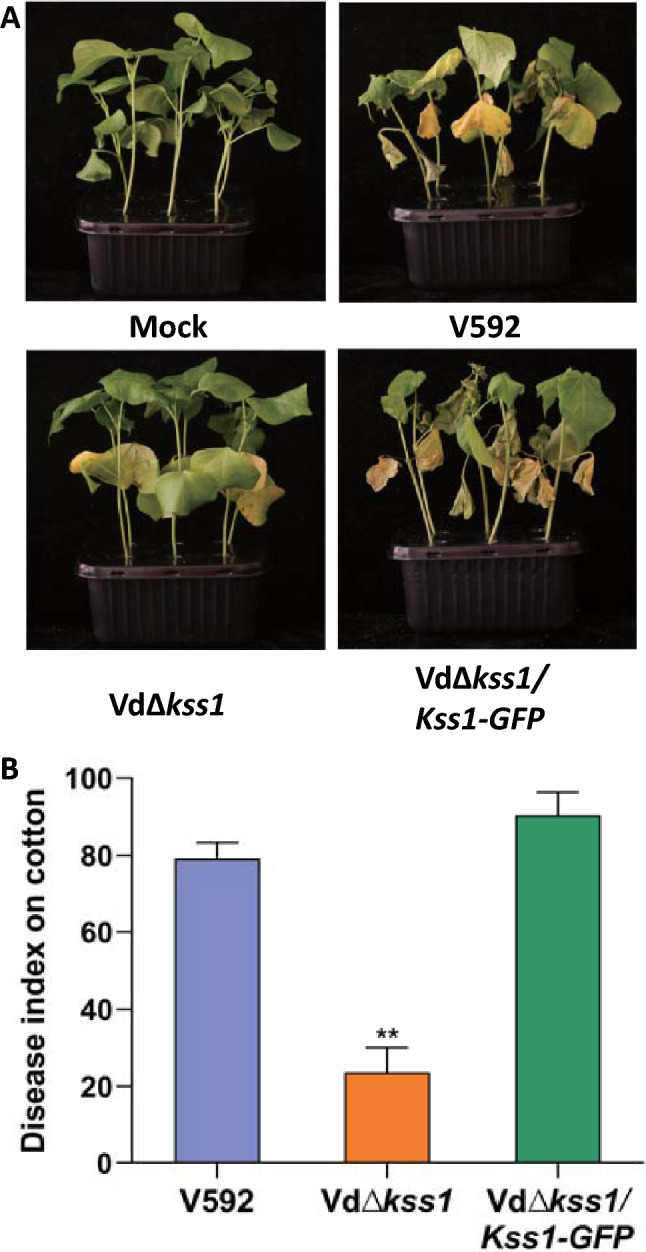
VdKss1 contributes to *Verticillium dahliae* virulence in cotton plants. **A** Disease symptoms of upland cotton. Plants infected with indicated strains were photographed and subjected to disease index analyses 3–4 weeks post inoculation. **B** Disease index analyses of upland cotton infected with the indicated strains. The disease indexes were evaluated with three replicates generated from 24 plants for each inoculum. Error bars indicate the standard deviation of three biological replicates. Student’s *t*-test was carried out to determine the significance of difference. **Indicates significant difference at *P*-value of < 0.01

The Vd*∆kss1* mutant and WT strains were tested for pathogenicity in cotton plants. The Vd*∆kss1* mutant displayed much less severe disease symptoms than the WT strain (Fig. 3A, B). The virulence of the Vd*∆kss1* mutant was restored upon complementation with *VdKss1* (Fig. 3A, B). These results indicate that *VdKss1* plays an important role in regulating the development of hyphopodium and is required for the full pathogenicity of *V. dahliae*.

### VdSte7 phosphorylates VdKss1 and regulates hyphopodium formation

**Fig. 4:**
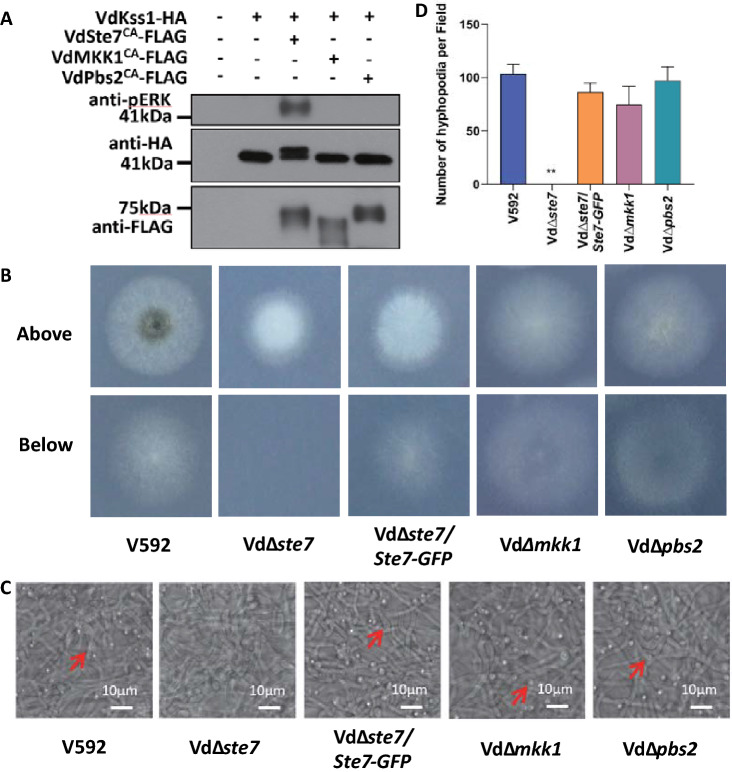
VdSte7 phosphorylates VdKss1 and regulates hyphopodium formation. **A** The constitutive active (CA) mutant VdSte7^CA^ specifically phosphorylates VdKss1. *Verticillium dahliae* protoplasts were transfected with VdKss1-HA alone or together with VdSte7^CA^-FLAG, VdMKK1^CA^-FLAG, or VdPbs2^CA^-FLAG. Proteins were extracted 16 h post transfection and subjected to Co-immunoprecipitation, then followed by anti-pERK or anti-HA immunoblot. The experiments were repeated three times with similar results. **B** Penetration phenotypes of *VdMAPKKs* deletion mutants and Vd∆*ste7*/*VdSte7-GFP* complementary strain. The spores of indicated strains were grown on MM covered with a cellophane for 5 days and photographed (above). The cellophane was removed and the culture was continued for 3 days and photographed (below). **C** Hyphopodium formation of *VdMAPKKs* deletion mutants and Vd∆*ste7*/*VdSte7-GFP* complementary strain. Indicated strains were grown on MM covered with a cellophane for 5 days. Hyphopodium formation (red arrow) on the cellophane was observed by confocal laser scanning microscopy (CLSM). **D** Hyphopodium quantification of *VdMAPKKs* deletion mutants and Vd∆*ste7*/*VdSte7-GFP* complementarystrain. The number of hyphopodium in three fields was counted for each strain. Error bars indicate the standard deviation of three fields. Student’s *t*-test was carried out to determine the significance of difference. **Indicates significant difference at *P-*value of < 0.01

Within the MAPK cascade, MAPKKK, MAPKK, and MAPK are sequentially phosphorylated. To determine the upstream MAPKKs responsible for phosphorylating VdKss1, *V. dahliae MAPKK* genes were cloned and analyzed for their ability to activate VdKss1. The genome of *V. dahliae* contains three putative MAPKK genes, *VdSte7* (VDAG_08626), *VdMKK1* (VDAG_09823), and *VdPbs2* (VDAG_02783) (Hamel et al. 2012). Double mutation of the conserved serine and/or threonine residues located in the kinase-activation loop converts MAPKKs into their constitutive active (CA) forms (Asai et al. 2002; Liu and Zhang 2004; Ren et al. 2002). CA mutants of each of the above MAPKKs were generated and co-expressed with VdKss1 in *V. dahliae* protoplasts. The phosphorylation of VdKss1 TEY motif was examined using an anti-pERK immuno-blot. VdSte7^CA^, but not VdMKK1^CA^ or VdPbs2^CA^, specifically induces the phosphorylation of VdKss1 (Fig. 4A), indicating that VdSte7 can phosphorylate VdKss1.

We next generated mutants with gene deletion of individual *MAPKK* genes (Fig. S5) and examined the penetration and development of hyphopodium in these mutants. Consistent with the specific phosphorylation of VdKss1 induced by VdSte7^CA^, the Vd*∆ste7* mutant, but neither the Vd*∆mkk1* nor the Vd*∆pbs2* mutants, exhibited compromised membrane penetration (Fig. 4B) and significantly reduced hyphopodium formation (Fig. 4C, D). The complementation of *VdSte7* in the Vd∆*ste7* mutant restored membrane penetration in the medium (Fig. 4B) and hyphopodium formation (Fig. 4C, D). Expression of VdSte7-GFP in the complementary strain was confirmed by anti-GFP immunoblot (Fig. S4B). The results indicating that VdSte7 acts as the upstream MAPKK to phosphorylate VdKss1 and contributes to the development of hyphopodium in *V. dahliae*.

### VdSte11 activates VdKss1 and regulates hyphopodium formation

**Fig. 5:**
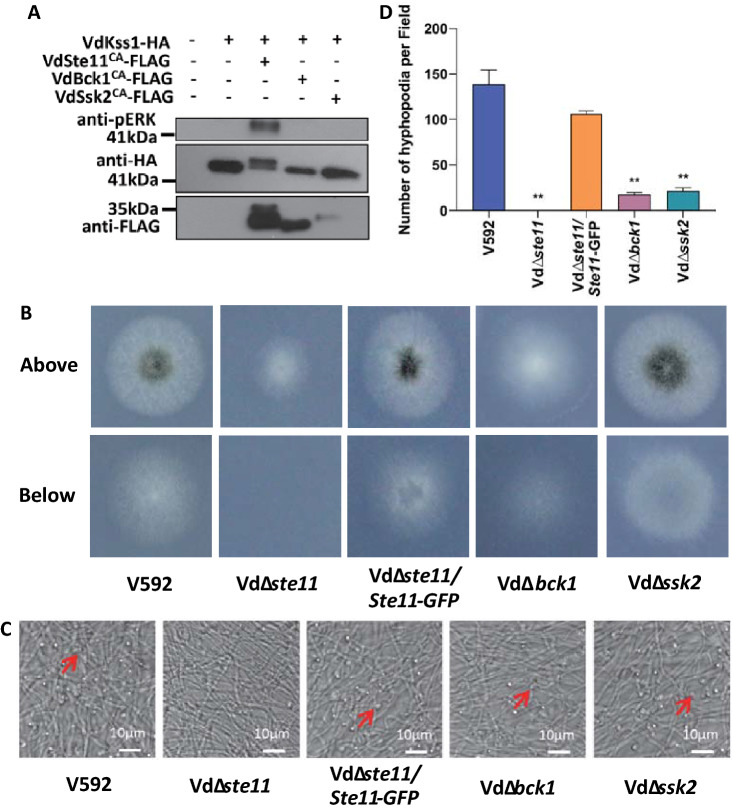
VdSte11 activates VdKss1 and regulates hyphopodium formation. **A** The CA mutant VdSte11^CA^ activates VdKss1. *Verticillium dahliae* protoplasts were transfected with VdKss1-HA alone or together with VdSte11^CA^-FLAG, VdBck1^CA^-FLAG, or VdSsk2^CA^-FLAG. Proteins were extracted 16 h post transfection and subjected to Co-immunoprecipitation, then followed by anti-pERK or anti-HA immunoblot. The experiments were repeated three times with similar results. **B** Penetration phenotypes of *VdMAPKKKs* deletion mutants and Vd∆*ste11*/*VdSte11-GFP* complementary strain. The spores of indicated strains were grown on MM covered with a cellophane for 5 days and photographed (above). The cellophane was removed and the culture was continued for 3 days and photographed (below). **C** Hyphopodium formation of *VdMAPKKKs* deletion mutants and Vd∆*ste11*/*VdSte11-GFP* complementary strain. Indicated strains were grown on MM covered with a cellophane for 5 days. Hyphopodium formation (red arrow) on the cellophane was observed by confocal laser scanning microscopy (CLSM). **D** Hyphopodium quantification of *VdMAPKKKs* deletion mutants and Vd∆*ste11*/*Ste11-GFP* complementary strain. The number of hyphopodium in three fields was counted for each strain. Error bars indicate the standard deviation of three fields. Student’s *t*-test was carried out to determine the significance of difference. **Indicates significant difference at *P-*value of < 0.01

VdSte11 (VDAG_05822), VdBck1 (VDAG_00874), and VdSsk2 (VDAG_08787) are three putative MAPKKKs encoded by *V. dahliae* (Hamel et al. 2012)*.* MAPKKK usually contains an N-terminal auto-inhibitory domain that negatively regulates kinase activity. Removal of the N-terminal inhibitory domain results in activation of MAPKKKs in the absence of upstream kinases (Asai et al. 2002; Bergmann et al. 2004). Deletion of *VdSte11*, a *V. dahliae* MAPKKK, has been reported to impair hyphopodium formation in *V. dahliae* (Yu et al. 2019), suggesting an important role of *VdSte11* in regulating *V. dahliae* hyphopodium development. Whether VdSte11 activates VdKss1 and whether additional MAPKKKs are involved in hyphopodium development remain undetermined. The truncated C-terminal forms of the individual MAPKKKs, VdSte11^CA^, VdBck1^CA^, and VdSsk2^CA^, were then constructed and co-expressed with VdKss1 in *V. dahliae* protoplasts. Co-expression of VdSte11^CA^, but not VdBck1^CA^ or VdSsk2^CA^, induced phosphorylation of VdKss1 (Fig. 5A), indicating that VdSte11 acts as the upstream MAPKKK that induces VdKss1 phosphorylation.

To further examine the role of MAPKKKs in the regulation of hyphopodium development, mutants with deletion of individual MAPKKKs were generated (Fig. S5). While the Vd*∆bck1* and Vd*∆ssk2* mutants exhibited normal penetration, the Vd*∆ste11* mutant exhibited deficient cellophane penetration (Fig. 5B). Expression of VdSte11-GFP in the complementary strain was confirmed by anti-GFP immunoblot (Fig. S4C). All three mutants generated fewer hyphopodia than the WT strains, among which the Vd*∆ste11* mutant was almost impaired in hyphopodium formation (Fig. 5C, D). The results indicated that VdSte11, VdBck1, and VdSsk2 are all involved in regulating hyphopodium formation, and that VdSte11 plays a greater role than VdBck1 and VdSsk2.

### VdSte7 and VdSte11 are required for full pathogenicity of *V. dahliae*

**Fig. 6:**
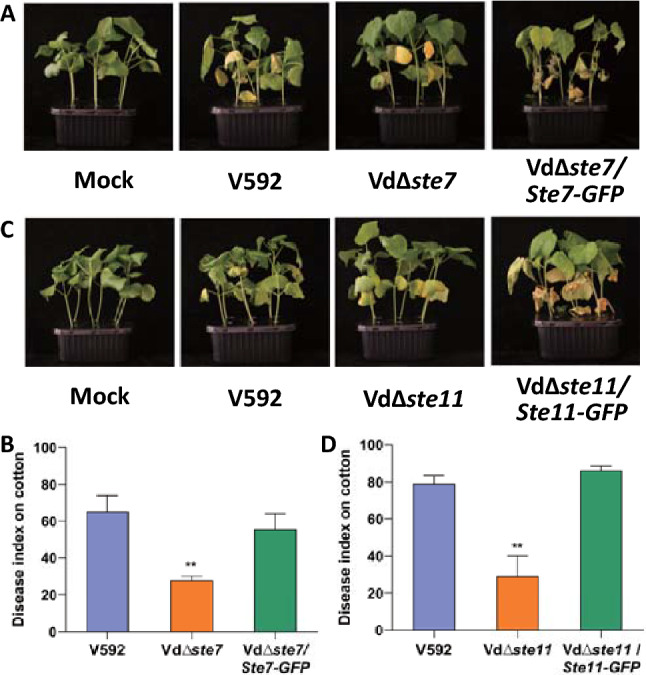
Deletion of *VdSte7* or *VdSte11* compromises virulence of *Verticillium dahliae* in cotton plants. **A, C**. Disease symptoms of upland cotton. Plants infected with indicated strains were photographed and subjected to disease index analyses 3–4 weeks post inoculation. **B, D** Disease index analyses of upland cotton infected with the indicated strains. The disease indexes were evaluated with three replicates generated from 24 plants for each inoculum. Error bars indicate the standard deviation of three biological replicates. Student’s *t*-test was carried out to determine the significance of difference. **Indicates significant difference at *P*-value of < 0.01

To determine the role of VdSte7 and VdSte11 in pathogenicity, Vd*∆ste7*, Vd*∆ste11* mutants and WT strains were subjected to pathogenicity assays in cotton plants. Reduced pathogenicity was observed in both the Vd*∆ste7* (Fig. 6A, B) and Vd*∆ste11* mutants (Fig. 6C, D). Complementation of *VdSte7* in Vd*∆ste7* mutant (Fig. 6A, B) and *VdSte11* in Vd*∆ste11* (Fig. 6C, D) restored their pathogenicity to the level of the WT strain. These results suggest that both VdSte7 and VdSte11 are required for full pathogenicity of *V. dahliae.* Thus, the results indicate that VdSte11-VdSte7-VdKss1 constitutes a complete MAPK cascade that regulates the hyphopodium formation and pathogenicity of *V. dahliae.*

## Discussion

*V. dahliae* infects more than 200 kinds of plants, causing *Verticillium* wilt, leading to severe yield losses worldwide. Formation of hyphopodium is crucial for the establishment of *V. dahliae* infection, and the underlying regulatory mechanisms remain poorly characterized. We have previously reported the cAMP-mediated regulation of hyphopodium formation (Sun et al. 2019). VdSho1, a tetraspan transmembrane protein, regulates *V. dahliae* cellophane penetration and virulence in plants via the downstream MAPK signaling adaptor Vst50 (Li et al. 2019). *Saccharomyces cerevisiae* transmembrane mucin Msb2, which is widely conserved in fungi, functions upstream of the Kss1 MAPK cascade to regulate filamentous growth (Cullen et al. 2004; Perez-Nadales and Di Pietro 2011). In *V. dahliae*, VdMsb, has been reported to be required for plant infection and microsclerotia formation (Jiang et al. 2018; Tian et al. 2014). Moreover, mutation of *VdSte11* has also been shown to impair hyphopodium formation (Yu et al. 2019), indicating the involvement of MAPK cascades in regulating hyphopodium formation. However, a complete MAPK cascade has not been characterized yet.

In this study, we explored the function of the MAPK pathway and identified VdSte11-VdSte7-VdKss1 as a complete MAPK cascade that regulates *V. dahliae* hyphopodium formation and virulence*.* Among the five MAPKs encoded by *V. dahliae*, VdKss1 and VdHog1-1 have been shown to regulate microsclerotia formation and pathogenicity (Rauyaree et al. 2005; Wang et al. 2016b). Our study indicated that VdKss1, but not VdHog1-1, is essential for hyphopodium formation. In addition, VdBck1 and VdSsk2 are involved in hyphopodium formation*.* However, the CA forms of VdBck1 and VdSsk2 did not induce VdKss1 phosphorylation, suggesting the existence of additional regulatory mechanisms of hyphopodium formation that are mediated by VdBck1 and VdSsk2.

Although MAPK cascades are highly conserved in fungi, differential MAPK cascade specificities also occurs in different fungi. In *S. cerevisiae*, the high osmolarity glycerol (HOG) pathway activates the Ste11/Ssk2/Ssk22-Pbs2-Hog1 MAPK cascades (Hamel et al. 2012). In *V. dahliae*, however, VdSsk2, but not VdSte11, induces the phosphorylation of VdHog1 in response to stress. Also, a differential contribution between VdSsk2 and VdSte11 in *V. dahliae* pathogenesis has been reported (Yu et al. 2019).

The MAPK cascade regulating *V. dahliae* hyphopodium formation is highly homologous to that utilized by *M. oryzae* in the regulation of appressorium formation. Hog homologs are widely found in fungi including *M. oryzae* (Dixon et al. 1999), *Bipolaris oryzae* (Moriwaki et al. 2006), *Botrytis cinerea* (Segmuller et al. 2007), and the oomycete *Phytophthora sojae* (Li et al. 2010). Hog pathway is vital for the accumulation of osmo-protectant molecules and thus required for responses to environmental stresses in fungi (Hamel et al. 2012). Hog pathway also regulates virulence in some phytopathogens but not in *M. oryzae* (Dixon et al. 1999; Wang et al. 2016b). Our study showed that VdHog1-1 and VdHog1-2 are dispensable for hyphopodium formation in *V. dahliae*. The evidence indicates the convergence of MAPK cascades in *M. oryzae* and *V. dahliae*, although different infection-related structures are developed by the two pathogens during infection.

In phytopathogenic fungi *M. oryzae*, the appressorium formation is regulated by the conserved MAPK pathway Mst11-Mst7-Pmk1. As the major intracellular MAPK that is targeted by HopAI in *M. oryzae*, Mps1 (the ortholog of Slt2 in *S. cerevisiae*) is important for appressorium penetration and plant infection but is not necessary for appressorium formation (Xu et al. 1998; Zhang et al. 2017). In *M. oryzae*, G protein-coupled receptors (GPCRs) (Li et al. 2012, 2007b) and cyclic adenosine monophosphate (cAMP)-protein kinase A (PKA) signaling pathways are involved in the regulation of appressoria formation (Jin et al. 2013; Zhao et al. 2007).

We have showed that *VdKss1* plays an important role in regulating the development of hyphopodium and is required for the full pathogenicity of *V. dahliae*. In addition, the *VdSlt2* also plays an important role in hyphopodium formation and pathogenicity of *V. dahliae*. Our results suggested that both *VdKss1* and *VdSlt2* contribute to *V. dahliae* hyphopodium formation and pathogenicity, with *VdKss1* plays a greater role. Whether and how GPCRs and cAMP pathways interplay with the VdSte11-VdSte7-VdKss1 cascade to regulate hyphopodium formation in *V. dahliae* remains to be further investigated.

## Supplementary Information

Below is the link to the electronic supplementary material.

Supplementary file1 (DOCX 1532 KB)

## Data Availability

All data generated or analyzed during this study are included in this published article and its supplementary information files.
