# Supplementary material for: A complete MAP kinase cascade controls hyphopodium formation and virulence of Verticillium dahliae
Source: aBIOTECH. 2023 May 2;4(2):97–107. doi: 10.1007/s42994-023-00102-y (PMC10423180; doi:10.1007/s42994-023-00102-y)
Supplement: Supplementary file 1 — Supplementary file1 (DOCX 1532 KB) [file 42994_2023_102_MOESM1_ESM.docx]

**Supplementary Information**

**A complete MAP kinase cascade controls hyphopodium formation and virulence of *Verticillium dahliae***

Ziqin Ye^1,2^, Jun Qin^3^, Yu Wang^1,2^, Jinghan Zhang^1,4^, Xiaoyun Wu^1,2^, Xiangguo Li^5^, Lifan Sun^1,2^ and Jie Zhang^1,2*^

^1^State Key Laboratory of Plant Genomics, Institute of Microbiology, Chinese Academy of Sciences, Beijing, 100101, China.

^2^CAS Center for Excellence in Biotic Interactions, University of Chinese Academy of Sciences, Beijing 100049, China.

^3^State Key Laboratory of Crop Stress Biology for Arid Areas, College of Plant Protection, Northwest A&F University, Yangling, Shaanxi 712100, China.

^4^School of Life Sciences, Hebei University, Baoding, Hebei 710023, China.

^5^College of Agronomy, Shanxi Agricultural University, Taigu, Shanxi 030801, China.

Correspondence: zhangjie@im.ac.cn (J. Zhang)

**
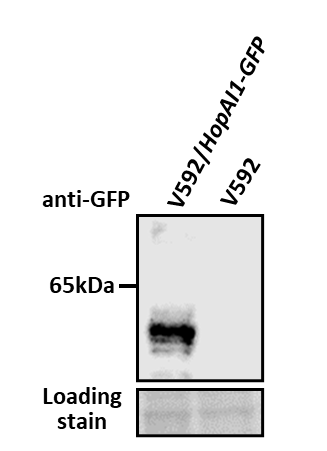
**

**Fig. S1 The expression of HopAI1-GFP protein in the *HopAI1*-expressing strain.**

The wild-type (WT) and *HopAI1*-expressing strains were cultured on the PDB medium for 3 days at 25℃. Total proteins were extracted and subjected to anti-GFP immunoblot.

**
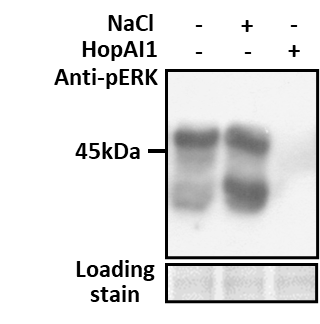
**

**Fig. S2** **MAPK activities are inhibited by HopAI1 in *V. dahliae*.**

The WT and *HopAI1*-expressing strains of *V. dahliae* were cultured on the PDA medium. After 5 days of culturation, the WT, salt stress-induced WT and *HopAI1*-expressing strains were cultured in the PDB medium supplied with or without 0.5M NaCl for 2 days at 25℃. Total proteins were extracted and subjected to anti-pERK immunoblot.

**
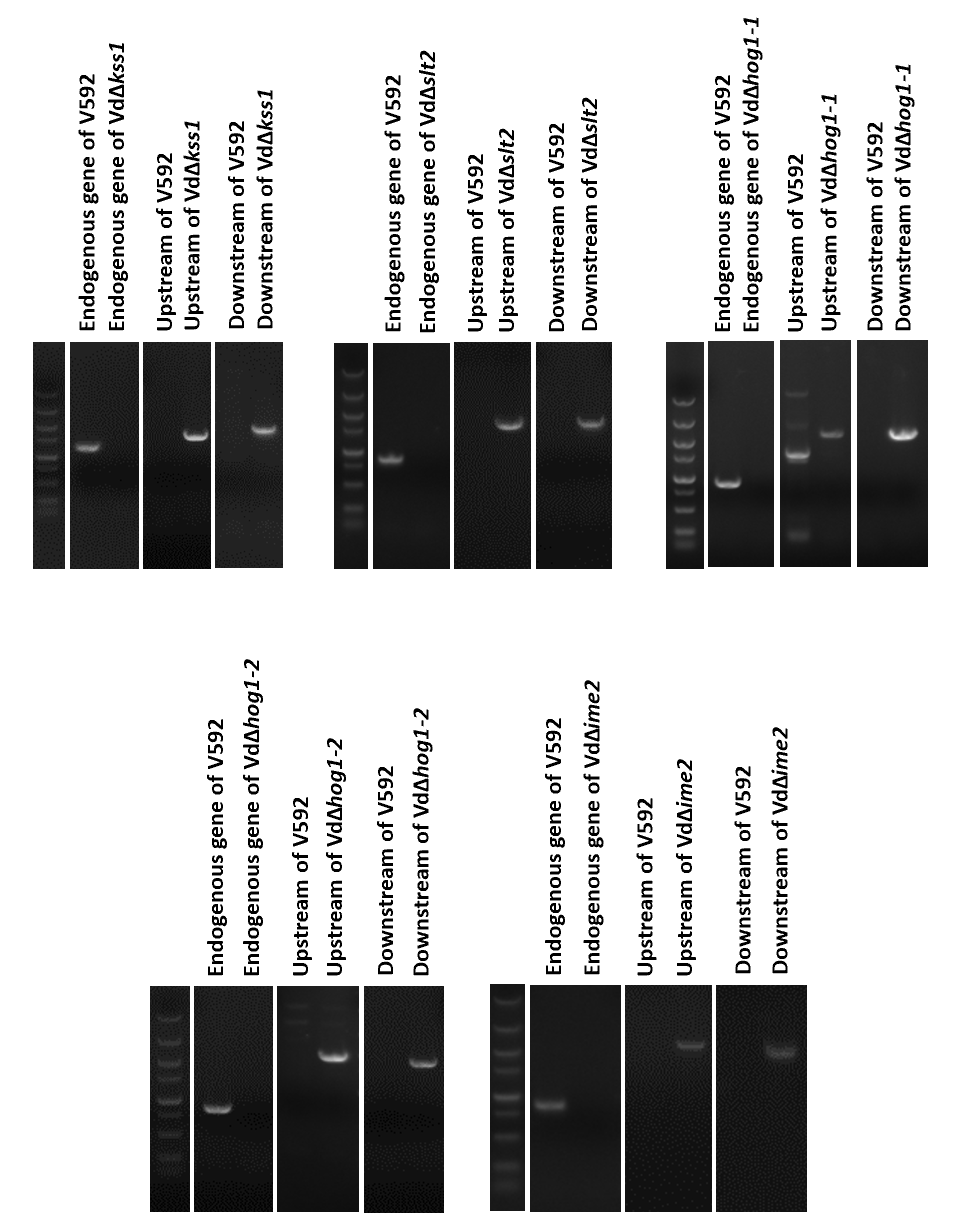
**

**Fig. S3 Verification of *VdMAPKs* deletions by PCR analyses.**

Target genes up 5’ flank-F and dn 3’ flank-R were paired with hygromycin B phosphotransferase (HPT) primers respectively to detect the replacement of target genes by HPT. PCR results of endogenous gene exhibited the deletion of target genes.

**
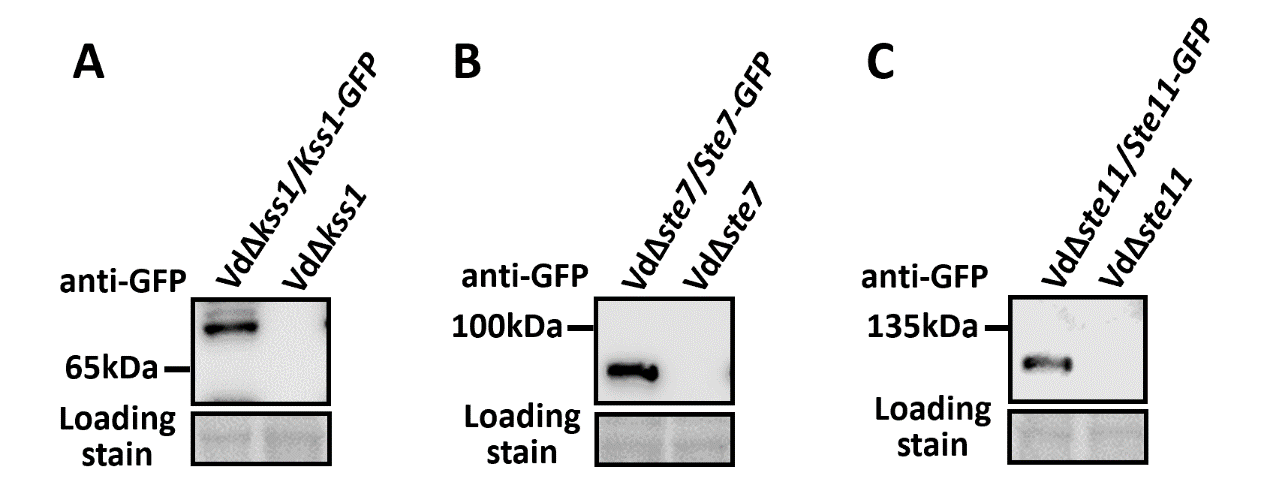
**

**Fig. S4 The expression of VdKss1-GFP, VdSte7-GFP or VdSte11-GFP proteins in complementary strains.**

The Vd∆*kss1*, Vd*∆ste7*, Vd∆*ste11* mutants and the corresponding complementary strains as indicated were cultured on the PDB medium for 3 days at 25℃. Total proteins were extracted and subjected to anti-GFP immunoblot.

**
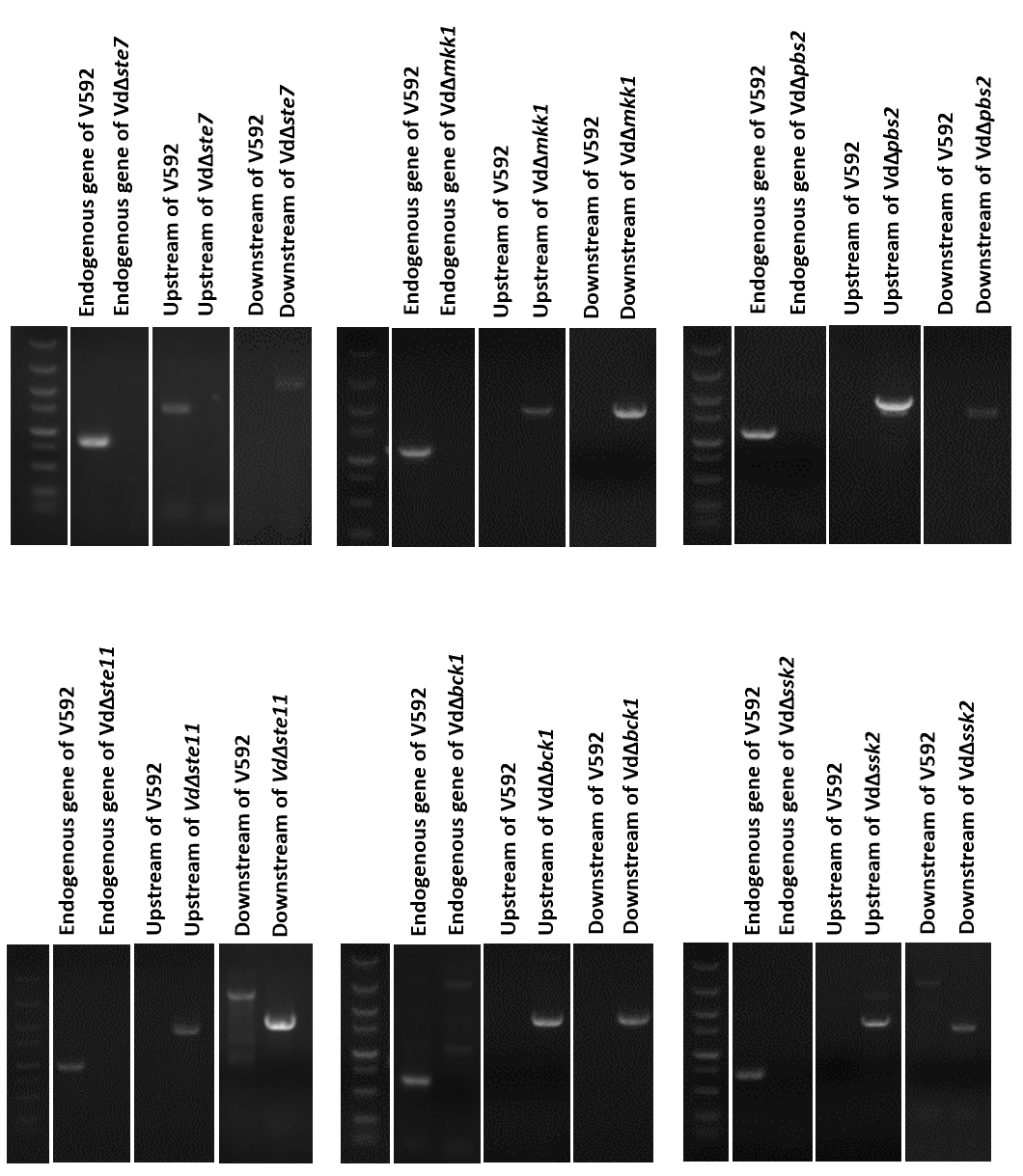
**

**Fig. S5 Verification of *VdMAPKKs* and *VdMAPKKKs* deletions by PCR analyses.**

Target genes up 5’ flank-F and dn 3’ flank-R were paired with hygromycin B phosphotransferase (HPT) primers respectively to detect the replacement of target genes by HPT. PCR results of endogenous gene exhibited the deletion of target genes.

**
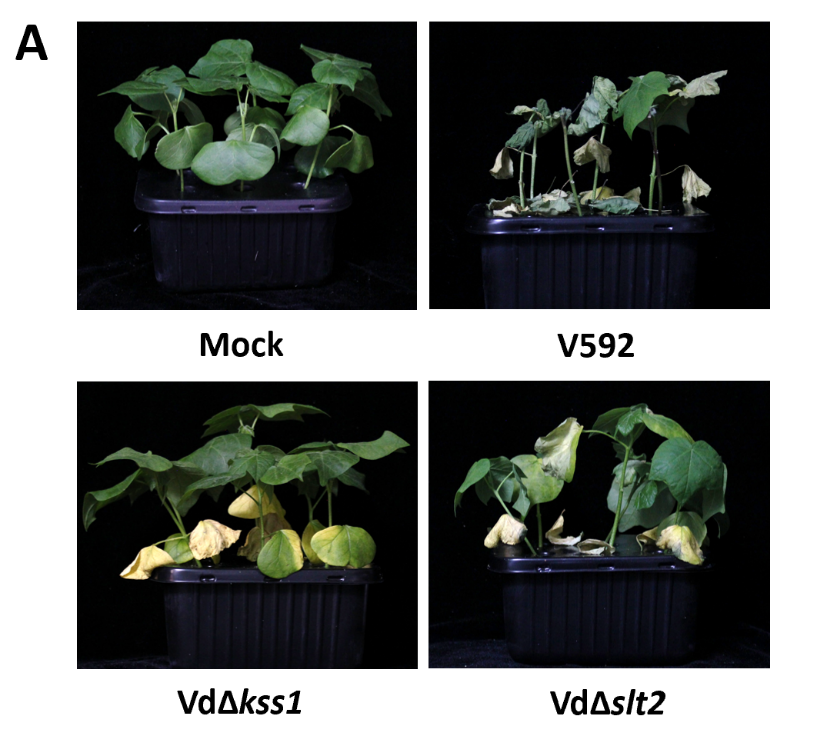
**

**
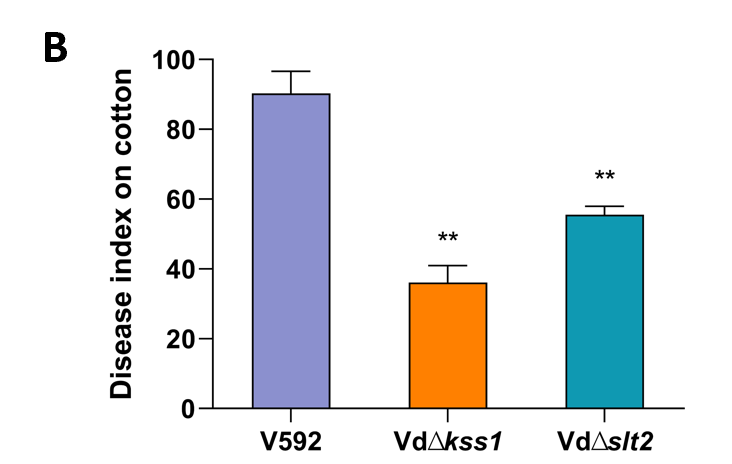
**

**Fig. S6 VdSlt2 and VdKss1 contribute to *Verticillium dahliae* virulence in cotton plants.**

**A** Disease symptoms of upland cotton plants infected with the indicated strains were photographed and subjected to disease index analyses 3 weeks post inoculation. **B** Disease index analyses of upland cotton infected with the indicated strains. The disease indexes were evaluated with three replicates generated from 18 plants for each inoculum. Error bars indicate the standard deviation of three biological replicates. Student’s *t*-test was carried out to determine the significance of difference. **Indicates significant difference at *P*-value of < 0.01.

**Table. S1** Primers used in this study

| **Primers** | **Sequence 5′-3′** | **Purpose** |
| --- | --- | --- |
| VdKss1-HA-F | 5’-ccgtcaaacggtaccatgtcgcgcagcaacgc-3’ | Protoplasts expression vector  construction |
| VdKss1-HA-R | 5’-gtatgggtagtcgacccgcataatctcctggtag-3’ |  |
| VdSte7-FLAG-F | 5’-ccgtcaaacggtaccatgcccgatcctttcgc-3’ |  |
| VdSte7-FLAG-R | 5’-tttgtagtcttcgaaattcgaggacccattgtaa-3’ |  |
| VdMKK1-FLAG-F | 5’-ccgtcaaacggtaccatggcggaccagaaccc-3’ |  |
| VdMKK1-FLAG-R | 5’-tttgtagtcttcgaacgtttccgcctttggcg-3’ |  |
| VdPbs2-FLAG-F | 5’-ccgtcaaacggtaccatggcagatctcaatacgc-3’ |  |
| VdPbs2-FLAG-R | 5’-tttgtagtcttcgaaggccagcgggctgcc-3’ |  |
| VdSte7^CA^-FLAG-F | 5’-ttgtcaacgatgtcgcagacgattttgttg-3’ | CA mutants protoplasts expression vector construction |
| VdSte7^CA^-FLAG-R | 5’-caacaaaatcgtctgcgacatcgttgacaa-3’ |  |
| VdMKK1^CA^-FLAG-F | 5’-ttggagacaagggcgaggccaacgagttca-3’ |  |
| VdMKK1^CA^-FLAG-R | 5’-tgaactcgttggcctcgcccttgtctccaa-3’ |  |
| VdPbs2^CA^-FLAG-F | 5’-tcgtcgcagacatggccaaggacaatattg-3’ |  |
| VdPbs2^CA^-FLAG-R | 5’-caatattgtccttggccatgtctgcgacga-3’ |  |
| VdSte11^CA^-FLAG-F | 5’-ccgtcaaacggtaccatgagcttccttgctggcgag-3’ |  |
| VdSte11^CA^-FLAG-R | 5’-tttgtagtcttcgaaggtaatgggcgccaggaagg-3’ |  |
| VdBck1^CA^-FLAG-F | 5’-ccgtcaaacggtaccatgaagcggcagacgacgttcc-3’ |  |
| VdBck1^CA^ -FLAG-R | 5’-tttgtagtcttcgaaaaactccttgccccgaatc-3’ |  |
| VdSsk2^CA^ -FLAG-F | 5’-ccgtcaaacggtaccatgtacctctcttcttcggcg-3’ |  |
| VdSsk2^CA^ -FLAG-R | 5’-tttgtagtcttcgaagtagaagccatcacccgtg-3’ |  |
| VdKss1-knockout-up-F | 5’-gagctcgctgagggtttaattaaaagcctggcctgtttcacg-3’ | Deletion vector construction |
| VdKss1-knockout-up-R | 5’-atgggcccgctgaggacttaattaaggttctggtacacgacgag-3’ |  |
| VdKss1-knockout-down-F | 5’-actagtgctgaggcattaattaaggtggcagtggcagtggcag-3’ |  |
| VdKss1-knockout-down-R | 5’-aagcttgctgaggtcttaattaacaggcagaaacgatcaacgg-3’ |  |
| VdSlt2-knockout-up-F | 5’-gagctcgctgagggtttaattaaactacgtcttcgccatggac-3’ |  |
| VdSlt2-knockout-up-R | 5’-atgggcccgctgaggacttaattaattcatagttagatgcggtcgg-3’ |  |
| VdSlt2-knockout-down-F | 5’-actagtgctgaggcattaattaagcagagcaaggaggaggatg-3’ |  |
| VdSlt2-knockout-down-R | 5’-aagcttgctgaggtcttaattaacccacacttcctgtcaacgg-3’ |  |
| VdHog1-1-knockout-up-F | 5’-gagctcgctgagggtttaattaagctacgacgtggcgaagtcg-3’ |  |
| VdHog1-1-knockout-up-R | 5’-atgggcccgctgaggacttaattaagtgggcgatgtgtggaaag-3’ |  |
| VdHog1-1-knockout-down-F | 5’-actagtgctgaggcattaattaatagagtgatgccacaggc-3’ |  |
| VdHog1-1-knockout-down-R | 5’-aagcttgctgaggtcttaattaaggcagggaggtcgtcagaac-3’ |  |
| VdHog1-2-knockout-up-F | 5’-gagctcgctgagggtttaattaacacggcgcgttcttgaatag-3’ |  |
| VdHog1-2-knockout-up-R | 5’-atgggcccgctgaggacttaattaactcgatggtttgaggatgtg-3’ |  |
| VdHog1-2-knockout-down-F | 5’-actagtgctgaggcattaattaaagaaatcccgtacggagcgtg-3’ |  |
| VdHog1-2-knockout-down-R | 5’-aagcttgctgaggtcttaattaagcctcgacaatacctcggtc-3’ |  |
| VdIme2-knockout-up-F | 5’-gagctcgctgagggtttaattaacgggttctgaaatctctgg-3’ |  |
| VdIme2-knockout-up-R | 5’-atgggcccgctgaggacttaattaattctgattgggaatctgacg-3’ |  |
| VdIme2-knockout-down-F | 5’-actagtgctgaggcattaattaaaggatgatttattttctgggag-3’ |  |
| VdIme2-knockout-down-R | 5’-aagcttgctgaggtcttaattaattggcgtaagtcatcttgcc-3’ |  |
| VdSte7-knockout-up-F | 5’-gagctcgctgagggtttaattaaagatcctcttaacggcgatg-3’ |  |
| VdSte7-knockout-up-R | 5’-atgggcccgctgaggacttaattaatttgggtgatgtgcgtggcc-3’ |  |
| VdSte7-knockout-down-F | 5’-actagtgctgaggcattaattaaacgccagcatccgaaacagc-3’ |  |
| VdSte7-knockout-down-R | 5’-aagcttgctgaggtcttaattaattaggcctgcttcgttggcc-3’ |  |
| VdMKK1-knockout-up-F | 5’-gagctcgctgagggtttaattaagttgccttcgcctcgcgaac-3’ |  |
| VdMKK1-knockout-up-R | 5’-atgggcccgctgaggacttaattaagacgtggttggcagcggtac-3’ |  |
| VdMKK1-knockout-down-F | 5’-actagtgctgaggcattaattaatgttgctcgacctgcagcgg-3’ |  |
| VdMKK1-knockout-down-R | 5’-aagcttgctgaggtcttaattaacagcagcccgacggacagac-3’ |  |
| VdPbs2-knockout-up-F | 5’-gagctcgctgagggtttaattaagttgagggcttctccaaactg-3’ |  |
| VdPbs2-knockout-up-R | 5’-atgggcccgctgaggacttaattaacttgcctgtcgagcaatgtg-3’ |  |
| VdPbs2-knockout-down-F | 5’-actagtgctgaggcattaattaaaagaagccatcgatattgcag-3’ |  |
| VdPbs2-knockout-down-R | 5’-aagcttgctgaggtcttaattaactaacgcgtaccagcgccacc-3’ |  |
| VdSte11-knockout-up-F | 5’-gagctcgctgagggtttaattaaaggttgactgatctacagg-3’ |  |
| VdSte11-knockout-up-R | 5’-atgggcccgctgaggacttaattaagttgacgatgtggcgatgag-3’ |  |
| VdSte11-knockout-down-F | 5’-actagtgctgaggcattaattaaatggcccctgaggcgcgag-3’ |  |
| VdSte11-knockout-down-R | 5’-aagcttgctgaggtcttaattaacgaggtcgaagacgacg-3’ |  |
| VdBck1-knockout-up-F | 5’-gagctcgctgagggtttaattaagtgtggaatgtatggatgcgg-3’ |  |
| VdBck1-knockout-up-R | 5’-atgggcccgctgaggacttaattaaggcgaagaattagtcgtgc-3’ |  |
| VdBck1-knockout-down-F | 5’-actagtgctgaggcattaattaagattgtgctgggctgcgagc-3’ |  |
| VdBck1-knockout-down-R | 5’-aagcttgctgaggtcttaattaacactggcctagcgacactgg-3’ |  |
| VdSsk2-knockout-up-F | 5’-gagctcgctgagggtttaattaaagcaacagacgacatctag-3’ |  |
| VdSsk2-knockout-up-R | 5’-atgggcccgctgaggacttaattaagccggctgcttttgatgtgc-3’ |  |
| VdSsk2-knockout-down-F | 5’-actagtgctgaggcattaattaagcgcacaagccctccttgtc-3’ |  |
| VdSsk2-knockout-down-R | 5’-aagcttgctgaggtcttaattaacggatcggaatcggcagagg-3’ |  |
| VdKss1 up 5’ flank-F | 5’-aagcctggcctgtttcacg-3’ | Verification of deletions |
| VdKss1 dn 3’ flank-R | 5’-gcgagacgggcttcctcag-3’ |  |
| VdKss1 endogenous gene F | 5’-acggtgtcgtctggtatgtc-3’ |  |
| VdKss1 endogenous gene R | 5’-agtcctccatcgtcggcg-3’ |  |
| VdSlt2 up 5’ flank-F | 5’-ctccgactttgacgactttg-3’ |  |
| VdSlt2 dn 3’ flank-R | 5’-ggaagagcaatatgtagatgac-3’ |  |
| VdSlt2 endogenous gene F | 5’-accaagacttcatcgtcgac-3’ |  |
| VdSlt2 endogenous gene R | 5’-tgtagctctggaagctcagc-3’ |  |
| VdHog1-1 up 5’ flank-F | 5’-gcccgagctgtcgtctgc-3’ |  |
| VdHog1-1 dn 3’ flank-R | 5’-agcttgccatgtaattctcg-3’ |  |
| VdHog1-1 endogenous gene F | 5’-caagtgagggaatcgactaac-3’ |  |
| VdHog1-1 endogenous gene R | 5’-tcttctctagcaggtcgatg-3’ |  |
| VdHog1-2 up 5’ flank-F | 5’-ccagatccagcttcgtttcc-3’ |  |
| VdHog1-2 dn 3’ flank-R | 5’-cctccttcaatgcttgggtc-3’ |  |
| VdHog1-2 endogenous gene F | 5’-agccaatatggaacggtcac-3’ |  |
| VdHog1-2 endogenous gene R | 5’-gagagtctgcagcagcaag-3’ |  |
| VdIme2 up 5’ flank-F | 5’-gcactggcgactccaggc-3’ |  |
| VdIme2 dn 3’ flank-R | 5’-gcaggattggatcgacgg-3’ |  |
| VdIme2 endogenous gene F | 5’-tccgagacgaccgatatctc-3’ |  |
| VdIme2 endogenous gene R | 5’-ggagcttcgtactggtgtac-3’ |  |
| VdSte7 up 5’ flank-F | 5’-ggatgcttggtttggggtac-3’ |  |
| VdSte7 dn 3’ flank-R | 5’-catgagcgtcacattagtctg-3’ |  |
| VdSte7 endogenous gene F | 5’-actaagtactggttgtggctg-3’ |  |
| VdSte7 endogenous gene R | 5’-aagtcgcgaatcggaaatgtg-3’ |  |
| VdMKK1 up 5’ flank-F | 5’-ccagacccaacccgacgaag-3’ |  |
| VdMKK1 dn 3’ flank-R | 5’-gtcatgcgccacctcggccg-3’ |  |
| VdMKK1 endogenous gene F | 5’-atggcggaccagaacccatc-3’ |  |
| VdMKK1 endogenous gene R | 5’-tgagatcttgccgaggacct-3’ |  |
| VdPbs2 up 5’ flank-F | 5’-ctcgtctcgtgcagatggg-3’ |  |
| VdPbs2 dn 3’ flank-R | 5’-cttccttcctcgcctgctgc-3’ |  |
| VdPbs2 endogenous gene F | 5’-atggcagatctcaatacgcc-3’ |  |
| VdPbs2 endogenous gene R | 5’-cgccctcttggaagaaggcc-3’ |  |
| VdSte11 up 5’ flank-F | 5’-tcttgccgccgttcattg-3’ |  |
| VdSte11 dn 3’ flank-R | 5’-gtacacggagcgcgacag-3’ |  |
| VdSte11 endogenous gene F | 5’-cagctccaacatggtcgcc-3’ |  |
| VdSte11 endogenous gene R | 5’-gcgtagtgtgacacgcatg-3’ |  |
| VdBck1 up 5’ flank-F | 5’-gggagcgtggatgtgaatc-3’ |  |
| VdBck1 dn 3’ flank-R | 5’-cacctgaggaacagcgaca-3’ |  |
| VdBck1 endogenous gene F | 5’-ctacaacccaaagttccacg-3’ |  |
| VdBck1 endogenous gene R | 5’-catgggacgaggacgagac-3’ |  |
| VdSsk2 up 5’ flank-F | 5’-tcttatctgctcctcagacc-3’ |  |
| VdSsk2 dn 3’ flank-R | 5’-ccggcactgccaccactc-3’ |  |
| VdSsk2 endogenous gene F | 5’-ctccaacggcgaggaagac-3’ |  |
| VdSsk2 endogenous gene R | 5’-acatgccgtaccactcgag-3’ |  |
| VdKss1-complement-F | 5’-aacctctagaggatccatgtcgcgcagcaacgc-3’ | Complementation vector construction |
| VdKss1-complement-R | 5’-gcagcttctgcgaattcccgcataatctcctggtag-3’ |  |
| VdSte7-complement-F | 5’-aacctctagaggatccatgcccgatcctttcgc-3’ |  |
| VdSte7-complement-R | 5’-gcagcttctgcgaattcattcgaggacccattgtaa-3’ |  |
| VdSte11-complement-F | 5’-aacctctagaggatccatggccatgctggctcccaa-3’ |  |
| VdSte11-complement-R | 5’-gcagcttctgcgaattcggtaatgggcgccaggaaggg-3’ |  |
